# The use of dried blood spot sampling for the measurement of HbA1c: a cross-sectional study

**DOI:** 10.1186/s12907-015-0013-5

**Published:** 2015-07-08

**Authors:** Claudio A. Mastronardi, Belinda Whittle, Robert Tunningley, Teresa Neeman, Gilberto Paz-Filho

**Affiliations:** Department of Genome Sciences, The John Curtin School of Medical Research, The Australian National University, 131 Garran Rd, Canberra, Acton ACT 2601 Australia; Australian Phenomics Facility, The Australian National University, 117 Garran Rd, Canberra, Acton ACT 2601 Australia; Statistical Consulting Unit, The Australian National University, 27 Union Lane, Canberra, Acton ACT 2601 Australia

**Keywords:** Diabetes, Dried blood spot testing, HbA1c, Hemoglobin A1c, Turbidimetry

## Abstract

**Background:**

The use of dried blood spot (DBS) sampling is an alternative to traditional venous blood collection, and particularly useful for people living in rural and remote areas, and for those who are infirm, house-bound or time-poor. The objective of this study was to assess whether the measurement of glycated haemoglobin A1c (HbA1c) in DBS samples provided comparative and acceptably precise results.

**Methods:**

Venous and capillary blood samples were collected from 115 adult participants. After proper instruction, each participant punctured his/her own finger and collected capillary blood samples on pieces of a proprietary cellulose filter paper. Each filter paper was subsequently placed inside a breathable envelope, stored at room temperature, and processed on the same day (D0), four (D4), seven (D7) and fourteen (D14) days after collection. HbA1c was measured in duplicates/triplicates in whole venous blood (WB), capillary blood (capDBS) and venous blood placed on the matrix paper (venDBS), by turbidimetric inhibition immunoassay. Intra-assay coefficients of variation (CV) were calculated. DBS values were compared to WB results using linear regression, Bland-Altman plots and cross-validation models.

**Results:**

Eleven and 56 patients had type 1 and type 2 diabetes mellitus, respectively. Mean HbA1c levels were 6.22 ± 1.11 % for WB samples (n = 115). The median intra-assay CV was lower than 3 % for WB and capDBS on all days. Results from capDBS and venDBS showed high correlation and agreement to WB results, with narrow 95 % limits of agreement (except for results from D14 samples), as observed in Bland-Altman plots. When capDBS values were applied to equations derived from regression analyses, results approached those of WB values. A cross-validation model showed that capDBS results on D0, D4 and D7 were close to the WB results, with prediction intervals that were narrow enough to be clinically acceptable.

**Conclusions:**

The measurement of HbA1c from DBS samples provided results that were comparable to results from WB samples, if measured up to seven days after collection. Intra-assay coefficients of variation were low, results were in agreement with the gold-standard, and prediction intervals were clinically acceptable. The measurement of HbA1c through DBS sampling may be considered in situations where traditional venipuncture is not available.

**Trial registration:**

Australian New Zealand Clinical Trials Registry ID ACTRN12613000769785.

## Background

Glycated haemoglobin (HbA1c) is a biomarker that is fundamental for the diagnosis of diabetes and for monitoring glycaemic control [1]. Traditionally, its measurement depends on venipuncture, and on processing, transportation and storage of whole blood (WB) samples, which can be logistically challenging [2]. These challenges can sometimes compromise proper diagnosis and treatment of patients with diabetes mellitus.

An alternative blood sampling method, based on the use of a dry matrix, was first described in the literature over a century ago [3], and subsequently applied in the clinics to detect metabolic defects through the collection of heel capillary blood samples from newborns [4]. This method is centred on collecting blood samples obtained from finger or heel puncturing on a matrix paper, which is subsequently dried. These dried blood spots (DBS) can then be used for the measurement of diverse substances, including HbA1c, and requires minimal training of staff, is cheaper and safer, eliminates the need for special transportation logistics, and is more acceptable to study participants [5–7].

DBS sampling has been routinely and successfully used for the screening of congenital metabolic and endocrine diseases, such as phenylketonuria and hypothyroidism [8]. More recently, studies have shown that measurements of inflammatory markers, cytokines, serum antibodies, human immunodeficiency virus (HIV) loads and blood hormone levels provide results that are comparable to those obtained from standard venous samples [5, 9–11].

Dried blood spot sampling is also useful for the measurement of HbA1c in individuals with and without diabetes. A recent meta-analysis of seventeen heterogeneous studies demonstrated that HbA1c results from DBS were correlated to those obtained through venipuncture [12]. However, there is still a need for standardisation of sample collection, transportation, storage and analysis. In this study, we evaluated HbA1c levels collected on a novel matrix paper and measured through immunoturbidity, up to 14 days after DBS collection. Subsequently, to demonstrate whether DBS provide results comparable to WB samples, DBS results were compared against those obtained from standard methods.

## Methods

This study was approved by the Australian National University Human Research Ethics Committee, and all participants provided informed consent. We recruited participants from the general population living within the Australian Capital Territory region, Australia. Inclusion criteria allowed all adults over 18 years-old from all genders and ethnicities that had no restrictions to having their blood drawn (*i.e.* due to religious matters, or blood donation in the previous 4 weeks, or difficulty in providing venous blood samples). Participants were advised not to consume food, alcohol or caffeine for 12 h prior to the collection.

Venous blood was collected from an arm vein following standard sterile techniques, into EDTA-coated plastic tubes, providing WB samples. For the collection of capillary DBS (capDBS) samples, we used a 2 x 3 inch dry matrix cellulose paper with nine 10-mm outer diameter circles printed on the surface (ITL Healthcare Pty Ltd). Each printed circle has the capacity to hold 30 to 40 microlitres of blood. Participants were instructed to collect their capillary blood through finger pricking and placing one drop of blood onto each of the pre-defined circles of the dry matrix paper, at room temperature (23 ° C). The matrix paper was then placed into a breathable envelope (ITL Healthcare Pty Ltd) for transportation to the testing laboratory, and blood spots were allowed to dry at room temperature for >2 h before transportation. Forty-microlitre drops of venous blood were also pipetted from collection tubes with no anticoagulant and immediately placed on another dry matrix card, providing venous DBS (venDBS) samples.

All blood samples were transported to the pathology laboratory for analysis. HbA1c levels were determined by a direct turbidimetric inhibition immunoassay that determines HbA1c as a percentage of total haemoglobin (%HbA1c) (Thermo Fisher Scientific). Assays were performed on an Indiko Plus (Thermo Fisher Scientific) automated biochemistry analyser, and results were reported as %HbA1c NGSP values.

Processing of DBS samples (capDBS and venDBS): For each participant sample, two punches were taken from one DBS near the outer edge of the spot. Each punch had 3.2 mm in diameter and contains approximately 1.4 μL of serum. Punches were placed in haemolysing reagent (Thermo Fisher Scientific), in duplicate or triplicate, and incubated at room temperature with shaking. For each duplicate, one milliliter of haemolysate was processed in the Indiko analyser as per the standard protocol for whole blood. capDBS and venDBS samples were processed and analysed on the same day (D0), and on D4, D7 and D14, in duplicates or triplicates for the calculation of intra-assay coefficients of variation (CV).

Processing of WB samples: WB samples were prepared and processed as per standard protocol (Thermo Fisher Scientific). WB samples were processed and analysed on the same day (D0), in duplicates.

Results were presented as mean ± SD or median and range. Linear regression models for predicting WB from DBS were fit and goodness-of-fit measures [mean standard error (MSE) and R-squared] were estimated using cross-validation (R program developed for the cross-validation available upon request). From these models, we predicted WB from DBS values of 4 %, 7 %, 7.5 % and 10 % and in addition, obtained 95 % prediction intervals for all days. DBS values were applied to equations derived from linear regression analyses from D0, D4, D7 and D14 data, in order to obtain corrected DBS values (*i.e.*, to bring uncorrected DBS values closer to the line of equality). Bland-Altman plots were constructed with corrected D0, D4, D7 and D14 results.

## Results

A total of 115 participants (n = 51 males, n = 64 females) were recruited. Mean age was 55.9 ± 15.3 years-old; 11 participants (9.6 %) had been previously diagnosed with type 1 diabetes, and 56 individuals (48.7 %) had type 2 diabetes. Overall mean whole blood HbA1c levels were 6.22 ± 1.11 % (5.41 ± 0.35 % for participants without diabetes, 7.80 ± 0.81 % for volunteers with type 1 diabetes, and 6.61 ± 1.11 % among individuals with type 2 diabetes). Characteristics of the studied participants are summarised in Table 1.

**Table 1:** Characteristics of the studied population

	All	No diabetes	Type 1 diabetes	Type 2 diabetes
Gender (Males:Females)	51 M:64 F	20 M:28 F	2 M:9 F	28 M:28 F
Age (years; mean ± SD)	55.9 ± 15.3	46.2 ± 14.4	45.0 ± 12.8	64.8 ± 10.0
WB HbA1c (%; mean ± SD)	6.22 ± 1.11 %	5.41 ± 0.35 %	7.80 ± 0.81 %	6.61 ± 1.11 %

Note: WB = whole blood; SD = standard deviation

Whole blood and dried blood spot samples (capillary and venous) were measured in duplicates or triplicates, allowing the determination of intra-assay CV. The median intra-assay CVs were 1.19 % for WB (range 0–4.1 %), and lower than 3 % for all other samples (Table 2).

**Table 2:** Summary of HbA1c results from WB, capillary DBS and venous DBS samples

Day	Sample	HbA1c (%, Mean ± SD)	Intra-assay CV % (median, range)
D0	WB	6.22 ± 1.11 (N = 115)	1.19, 0–4.10
	Uncorrected capDBS	6.62 ± 1.16 (N = 77)	2.28, 0–10.10
	Corrected capDBS	6.39 ± 1.17 (N = 77)	N/A
	Uncorrected venDBS	6.72 ± 1.20 (N = 81)	1.68, 0–6.86
	Corrected venDBS	6.42 ± 1.18 (N = 81)	N/A
D4	Uncorrected capDBS	6.92 ± 1.32 (N = 96)	2.28, 0–9.87
	Corrected capDBS	6.26 ± 1.18 (N = 96)	N/A
	Uncorrected venDBS	7.15 ± 1.39 (N = 81)	2.14, 0–11.79
	Corrected venDBS	6.42 ± 1.21 (N = 81)	N/A
D7	Uncorrected capDBS	6.85 ± 1.29 (N = 81)	1.98, 0–16.04
	Corrected capDBS	6.25 ± 1.15 (N = 81)	N/A
	Uncorrected venDBS	7.36 ± 1.47 (N = 75)	2.81, 0–26.42
	Corrected venDBS	6.51 ± 1.23 (N = 75)	N/A
D14	Uncorrected capDBS	6.62 ± 1.44 (N = 79)	2.62, 0–17.68
	Corrected capDBS	6.10 ± 1.33 (N = 79)	N/A
	Uncorrected venDBS	7.30 ± 1.65 (N = 79)	2.54, 0–26.52
	Corrected venDBS	6.44 ± 1.29 (N = 79)	N/A

Note: WB = whole blood; ven = venous; cap = capillary; DBS = dried blood spot; SD = standard deviation; CV = coefficient of variation; N/A = not applicable

Mean ± SD capillary DBS (capDBS) levels of HbA1c were 6.62 ± 1.16 % when measured on D0 (n = 77), 6.92 ± 1.32 % on D4 (n = 96), 6.85 ± 1.29 % on D7 (n = 81), and 6.62 ± 1.44 % on D14 (n = 79). Venous DBS (venDBS) samples ranged from 6.72 ± 1.20 % on D0 to 7.36 ± 1.47 % on D7. Mean capDBS and venDBS values were applied to correction formulas obtained from linear regression analyses for each day. Corrected DBS values were closer to WB results (except for D14). Table 2 summarizes the results from WB and DBS samples (corrected and uncorrected).

**Fig. 1:**
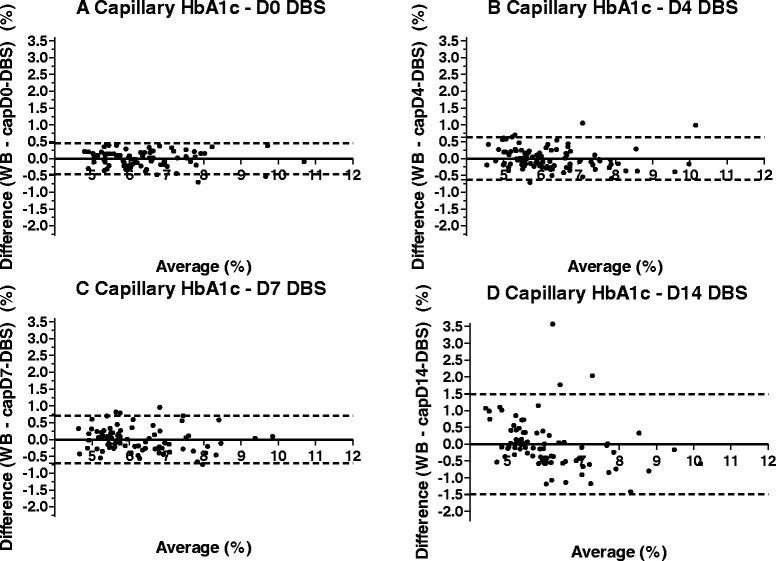
Bland-Altman plots of capillary dried blood spot samples from days 0, 4, 7 and 14. Note: Corrected DBS results are represented on D0, D4, D7 and D14; dashed lines represent 95 % limits of agreement; full lines represent biases. WB = whole blood on D0; cap = capillary; DBS = dried blood spot

**Fig. 2:**
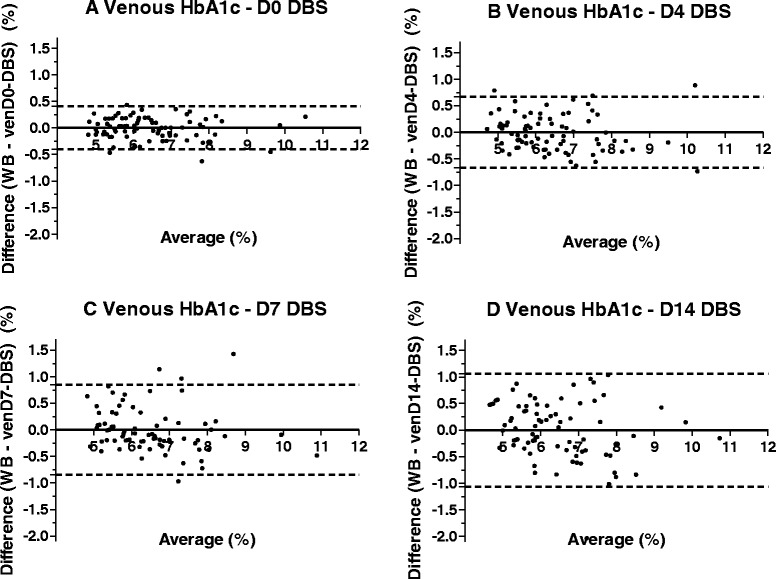
Bland-Altman plots of venous dried blood spot samples from days 0, 4, 7 and 14. Note: Corrected DBS results are represented on D0, D4, D7 and D14; dashed lines represent 95 % limits of agreement; full lines represent biases. WB = whole blood on D0; cap = capillary; DBS = dried blood spot

Bland-Altman plots of difference in HbA1c values in WB and corrected capDBS (Fig. 1), and in WB and corrected venDBS (Fig. 2) showed good correlation and agreement between the two methods, with few samples falling outside the 95 % limits of agreement for each comparison (average difference ± 1.96 standard deviation of the difference). However, limits of agreement were broader on D14 (Table 3).

**Table 3:** 95% limits of agreement for capillary and venous DBS results, uncorrected and corrected

		Upper limit		Lower limit	
		Uncorrected	Corrected	Uncorrected	Corrected
D0	Capillary	0.2308	0.462	−0.6828	−0.463
	Venous	0.1174	0.404	−0.7051	−0.402
D4	Capillary	0.1117	0.638	−1.428	−0.636
	Venous	0.1203	0.671	−1.567	−0.666
D7	Capillary	0.2383	0.668	−1.436	−0.668
	Venous	0.2345	0.845	−1.943	−0.845
D14	Capillary	1.111	1.484	−2.151	−1.485
	Venous	0.6642	1.065	−2.373	−1.069

From any given capDBS result, the linear regression models predicted WB values that were generally lower than the measured capDBS values. For example, for capDBS HbA1c results of 4 %, the predicted WB values were 3.84 % on Day 0, 3.86 % on Day 4, 3.97 % on Day 7, and 4.48 % on Day 14. For capDBS HbA1c results of 7 %, the predicted WB values were 6.76 % on Day 0, 6.33 % on Day 4, 6.37 % on Day 7, and 6.33 % on Day 14. For capDBS HbA1c results of 7.5 %, the predicted WB values were 7.25 % on Day 0, 6.73 % on Day 4, 6.77 % on Day 7, and 6.64 % on Day 14. For capDBS HbA1c results of 10 %, the predicted WB values were 9.69 % on Day 0, 8.79 % on Day 4, 8.78 % on Day 7, and 8.19 % on Day 14. The width of the 95 % prediction intervals (a measure of how precisely WB can be estimated) varied more broadly on D14. The estimated mean squared error (MSE) was lower on Days 0 and 4 when using the linear regression model, which also determined further decreases in R^2^ values on D7 and D14 (Table 4).

**Table 4:** Prediction intervals for WB from capillary DBS values of 4 %, 7 %, 7.5 % and 10 %, obtained through linear regression models

	capDBS 4 %			capDBS 7 %			capDBS 7.5 %			capDBS 10 %			MSE	Adjusted R^2^
	pWB	Lower 95 % CI	Upper 95 % CI	pWB	Lower 95 % CI	Upper 95 % CI	pWB	Lower 95 % CI	Upper 95 % CI	pWB	Lower 95 % CI	Upper 95 % CI		
D0	3.84	3.36	4.32	6.76	6.30	7.23	7.25	6.78	7.72	9.69	9.20	10.18	0.0554	0.9463
D4	3.86	3.22	4.51	6.33	5.70	6.95	6.74	6.11	7.36	8.79	8.14	9.43	0.0992	0.9064
D7	3.97	3.26	4.67	6.37	5.68	7.06	6.77	6.08	7.46	8.77	8.06	9.49	0.1247	0.8267
D14	4.48	3.20	5.76	6.33	5.08	7.59	6.64	5.38	7.90	8.19	6.89	9.48	0.3905	0.5268

Note: pWB = predicted whole blood; capDBS = capillary dried blood spot; MSE: mean standard error; CI: confidence interval

## Discussion

There is growing demand for human pathology test services in Australia and around the world, driven by the ageing global population and increasing incidence of chronic diseases [13]. Dried blood spot sampling is an alternative to traditional blood sampling, and has been used in clinical and epidemiological studies for several decades [6, 8, 14]. This method provides results that are comparable to those obtained through traditional venipucture [2, 12], without its logistical obstacles regarding sample collection, processing, transportation and storage. For the measurement of HbA1c, DBS has also been shown to produce results that are comparable to those obtained through venous sampling [15–25]. In our study, we showed that HbA1c levels from DBS samples collected via finger pricking from volunteers with and without diabetes were comparable to those measured from venous samples, when measured up to seven days after collection.

In our study, DBS samples collected from finger pricking (capDBS) were analysed on the same day (D0), four (D4), seven (D7) and fourteen (D14) days after collection. High correlation and agreement between capDBS results on D0 and venous blood HbA1c values showed that the analysis of samples collected on matrix paper and analysed immediately provides results that are similar to those obtained and processed by traditional methods.

In a real-life scenario, DBS samples are mailed or shipped to the pathology laboratory that performs the assays. Therefore, DBS samples are not analysed immediately. To assess whether this gap between collection and analysis may interfere with the results, we performed analyses also four, seven and fourteen days after collection. We observed that, over time, the correlation between DBS and venous blood results becomes weaker, and the 95 % limits of agreement become wider, especially for D14 results, which may be clinically unacceptable. It is noteworthy that WB samples also degrade over time when not analysed immediately, particularly if not kept refrigerated. Haemoglobin degradation products may show up in samples that have coagulated and aged. These products may co-elute with, or be incompletely separated from, HbA1c. In these cases, the HbA1c value obtained may be reported as higher than it actually is [26]. This effect is particularly evident for venDBS samples, which were collected without anticoagulant.

We used a linear regression model for cross-validation, to ensure unbiased measures of goodness-of-fit and prediction intervals for WB. In that model, capD0-DBS results were closer to the predicted WB values for all evaluated HbA1c steps (4 %, 7 %, 7.5 %, and 10 %), and the prediction intervals were narrower. On the remaining days, capDBS results were further away from the predicted WB values, and the prediction intervals broadened over time. In the clinics, capD0-DBS samples would provide the most accurate HbA1c results, closer to the predicted WB results and with a narrower prediction interval. However, the difference between predicted WB and both capD4- and capD7-DBS results may be clinically acceptable, as well as their prediction intervals. In the case of capD14-DBS results, their prediction intervals may be too wide to be clinically acceptable. We applied four different capDBS values to the model, but any result can be applied to it (R program available upon request), providing similar behaviour.

In some cases, patients may have difficulty in collecting sufficient amount of blood samples from finger pricking on the matrix paper. That difficulty was evidenced by the fact that the sample size for each day was not equal to the total number of recruited participants. Therefore, we assessed whether venous blood collected through standard methods and spotted on the matrix paper would produce similar results. In those analyses, venous DBS samples were correlated to traditionally-processed venous blood samples in a similar way as capillary DBS. Also, there was high correlation and agreement between capDBS and venDBS results.

In our study, we recruited 67 diabetic patients. Most of them had type 2 diabetes, and had HbA1c levels that are considered adequate (particularly among participants with type 2 diabetes). Only three participants had HbA1c levels higher than 9 %. Therefore, results might have been different should more participants with decompensated diabetes had been recruited. Samples were measured at least in duplicates, and the median intra-assay coefficients of variation were clinically acceptable, lower than 3 % at all times. However, some participants had heterogeneous results. It is unclear why results using the same sample and assay method may vary in some participants.

One of the key issues to be considered for the employment of DBS sampling is the standardization of the analysis of the DBS measurements. It is essential to predict, with the highest possible level of accuracy, the concentration of HbA1c in WB from the values measured in the DBS tests. In a recent study, a meta-analysis of seventeen heterogeneous studies (employing different methods for measuring HbA1c) was performed by Affan *et al.*, and a correction formula to approximate the DBS results to the WB values was published (12). We employed their correction formula in our current studies (results not presented), but the outcomes of the corrected DBS values resulted in a poorer approximation to the WB values. It appears that the time elapsed between sample collection and processing is a key component of the variability observed in the DBS sampling. Indeed, we found that there are significant differences among the Bland-Altman plots constructed from data on each particular day (*e.g.* D0, D4, D7 and D14) between DBS vs. WB. Thus, in our current analytical method, we analysed the data of each processed day independently by proposing mean values and prediction intervals for each particular processing day. Additionally, we corrected capillary DBS results by applying a correction formula that derived from regression analyses for each particular day, to approximate the regression line to the equality line. Thus, we obtained a different formula for each day, and observed that corrected capillary DBS results were closer to the predicted WB ones on all days except D14, when MSE was higher (*i.e.*, WB results were less precisely predicted).

We acknowledge that our study is limited by the fact that participants were evaluated in a controlled research setting, and results may be different when capillary HbA1c is evaluated in a real-life scenario. Future studies need to evaluate samples from participants who collect their capillary DBS samples on their own, and mail them to the testing laboratory via standard postal services (subjected to confounding factors such as delays and temperature variations). Furthermore, future studies should evaluate the prediction intervals for other elapsed times such as D1, D2, and D3, and also determine how these prediction intervals can be applied in the management of diabetes. To answer those questions, future studies should evaluate healthy individuals and those with diabetes who are treatment-naïve, and compare their DBS values and their prediction intervals with their WB HbA1c outcomes. By considering their WB values as the gold-standard, a more accurate clinical interpretation of the prediction intervals, as proposed here, could be established.

## Conclusion

In conclusion, HbA1c measured from DBS samples collected via finger pricking provided results that were comparable to those obtained from venous samples and measured by standard procedures. When results from DBS samples (processed up to 7 days after their collection) were applied to correction equations, HbA1c results with the most accuracy and the least clinically-acceptable variability were obtained, with high correlation and agreement to HbA1c results from whole venous blood, and with narrow 95 % limits of agreement. Those findings were further confirmed by a cross-validation model, which provided prediction intervals that were narrow enough to be clinically acceptable. In order for the measurement of HbA1c through DBS sampling to be considered in situations where traditional venipuncture is not available, further studies need to evaluate the effects of external factors, in a broader population.

## References

[1] American Diabetes A (2014). Standards of medical care in diabetes--2014. Diabetes Care.

[2] McDade TW (2014). Development and validation of assay protocols for use with dried blood spot samples. Am J Hum Biol.

[3] Bang I (1913). Ein verfahren zur mikrobestimmung von blutbestandteilen. Biochem Ztschr.

[4] Guthrie R, Susi A (1963). A Simple Phenylalanine Method for Detecting Phenylketonuria in Large Populations of Newborn Infants. Pediatrics.

[5] Mei JV, Alexander JR, Adam BW, Hannon WH (2001). Use of filter paper for the collection and analysis of human whole blood specimens. J Nutr.

[6] Parker SP, Cubitt WD (1999). The use of the dried blood spot sample in epidemiological studies. J Clin Pathol.

[7] Bhatti P, Kampa D, Alexander BH, McClure C, Ringer D, Doody MM, Sigurdson AJ (2009). Blood spots as an alternative to whole blood collection and the effect of a small monetary incentive to increase participation in genetic association studies. BMC Med Res Methodol.

[8] Wilcken B, Wiley V (2008). Newborn screening. Pathology.

[9] Corran PH, Cook J, Lynch C, Leendertse H, Manjurano A, Griffin J, Cox J, Abeku T, Bousema T, Ghani AC (2008). Dried blood spots as a source of anti-malarial antibodies for epidemiological studies. Malar J.

[10] Sherman GG, Stevens G, Jones SA, Horsfield P, Stevens WS (2005). Dried blood spots improve access to HIV diagnosis and care for infants in low-resource settings. J Acquir Immune Defic Syndr.

[11] Xu YY, Pettersson K, Blomberg K, Hemmila I, Mikola H, Lovgren T (1992). Simultaneous quadruple-label fluorometric immunoassay of thyroid-stimulating hormone, 17 alpha-hydroxyprogesterone, immunoreactive trypsin, and creatine kinase MM isoenzyme in dried blood spots. Clin Chem.

[12] Affan ET, Praveen D, Chow CK, Neal BC (2014). Comparability of HbA1c and lipids measured with dried blood spot versus venous samples: a systematic review and meta-analysis. BMC Clin Pathol.

[13] Britt H. An analysis of pathology test use in Australia. Australian Association of Pathology Practices Inc. 2008. Available from http://pathologyaustralia.com.au/wp-content/uploads/2013/03/DOD-paper-+-append.pdf. Accessed 7 May 2015.

[14] Williams SR, McDade TW (2009). The use of dried blood spot sampling in the national social life, health, and aging project. J Gerontol B Psychol Sci Soc Sci.

[15] Geethanjali FS, Kumar RS, Seshadri MS, Anjali (2007). Accuracy of filter paper method for measuring glycated hemoglobin. J Assoc Physicians India.

[16] Egier DA, Keys JL, Hall SK, McQueen MJ (2011). Measurement of hemoglobin A1c from filter papers for population-based studies. Clin Chem.

[17] Fokkema MR, Bakker AJ, de Boer F, Kooistra J, de Vries S, Wolthuis A (2009). HbA1c measurements from dried blood spots: validation and patient satisfaction. Clin Chem Lab Med.

[18] Gay EC, Cruickshanks KJ, Chase HP, Klingensmith G, Hamman RF (1992). Accuracy of a filter paper method for measuring glycosylated hemoglobin. Diabetes Care.

[19] Jeppsson JO, Jerntorp P, Almer LO, Persson R, Ekberg G, Sundkvist G (1996). Capillary blood on filter paper for determination of HbA1c by ion exchange chromatography. Diabetes Care.

[20] Jones TG, Warber KD, Roberts BD (2010). Analysis of hemoglobin A1c from dried blood spot samples with the Tina-quantR II immunoturbidimetric method. J Diabetes Sci Technol.

[21] Lacher DA, Berman LE, Chen TC, Porter KS (2013). Comparison of dried blood spot to venous methods for hemoglobin A1c, glucose, total cholesterol, high-density lipoprotein cholesterol, and C-reactive protein. Clin Chim Acta.

[22] Lakshmy R, Gupta R (2009). Measurement of glycated hemoglobin A1c from dried blood by turbidimetric immunoassay. J Diabetes Sci Technol.

[23] Little RR, McKenzie EM, Wiedmeyer HM, England JD, Goldstein DE (1986). Collection of blood on filter paper for measurement of glycated hemoglobin by affinity chromatography. Clin Chem.

[24] Lomeo A, Bolner A, Scattolo N, Guzzo P, Amadori F, Sartori S, Lomeo L (2008). HPLC analysis of HbA1c in dried blood spot samples (DBS): a reliable future for diabetes monitoring. Clin Lab.

[25] Wikblad K, Smide B, Bergstrom A, Wahren L, Mugusi F, Jeppsson JO (1998). Immediate assessment of HbA1c under field conditions in Tanzania. Diabetes Res Clin Pract.

[26] Selvin E, Coresh J, Jordahl J, Boland L, Steffes MW (2005). Stability of haemoglobin A1c (HbA1c) measurements from frozen whole blood samples stored for over a decade. Diabet Med.

